# Profile of children assisted in a teaching outpatient clinic of developmental disabilities in São Paulo, Brazil

**DOI:** 10.1590/1984-0462/2023/41/2022005

**Published:** 2023-05-29

**Authors:** Melina Alves da Frota, Rosa Miranda Resegue, Anete Colucci, Cecilia Micheletti

**Affiliations:** a Universidade Federal de São Paulo, São Paulo, SP, Brasil.

**Keywords:** Child development, Neurodevelopmental disorders, Intellectual disability, Developmental disabilities, Desenvolvimento infantil, Transtornos do neurodesenvolvimento, Deficiência intelectual, Deficiências do desenvolvimento

## Abstract

**Objective:**

To analyze the epidemiological and clinical profile of patients with developmental disabilities followed in a university clinic in Brazil.

**Methods:**

Descriptive, retrospective study, based on medical records. Children aged zero to 18 years with developmental problems, firstly evaluated between 2009 and 2018, were included. Patients with missing data or out of the age and time period established were excluded. There were nine losses and 374 patients constituted the final sample. Linear regression models were performed.

**Results:**

The mean age at the first assessment was 52.2±39.7 months and the age when the parents perceived the symptoms was 20.9±23.8 months. The most common impairment was motor associated with language delay (28.3%). The interval between the parents’ perception and the first consultation was associated with the mothers’ education and number of pregnancies. The age at first assessment was associated with the disability type. The number of pregnancies was associated with the child's age when the parents noticed the symptoms and at the first consultation.

**Conclusions:**

Parents’ recognition of the symptoms occurred early, however, there was a delay until the arrival at the clinic. Higher maternal education was associated with a shorter gap between perception of the developmental disability and consultation. A greater number of pregnancies was associated with a later perception of the developmental delay by the parents as well as a delay in the assessment and a wider interval between them. Motor problems were the most common in younger children, and language complaints in older ones.

## Resumo

**Objetivo:**

Estabelecer o perfil clínico-epidemiológico de pacientes com alterações do desenvolvimento acompanhados em uma clínica universitária brasileira.

**Métodos:**

Estudo descritivo, retrospectivo, baseado em prontuários. Foram incluídas crianças de zero a 18 anos com alterações do desenvolvimento, primeiramente avaliadas entre 2009 e 2018. Foram excluídos pacientes com dados ausentes ou fora da idade e do período estabelecidos. Houve nove perdas, e 374 pacientes constituíram a amostra final. Modelos de regressão linear foram executados.

**Resultados:**

A média de idade na primeira avaliação foi de 52,2±39,7 meses, e a média de idade quando os pais perceberam os sintomas foi de 20,9±23,8 meses. O comprometimento mais comum foi o motor associado ao da linguagem (28,3%). O intervalo entre a percepção dos pais e a primeira consulta esteve associado à escolaridade materna e ao número de gestações. A idade da primeira avaliação foi relacionada ao tipo de comprometimento. O número de gestações foi associado à idade da criança quando os pais notaram os sintomas e na primeira consulta.

**Conclusões:**

O reconhecimento dos sintomas pelos pais foi pontual, porém houve demora até a chegada à clínica. Maior escolaridade materna foi associada a menor defasagem entre a percepção da deficiência e a consulta. Maior número de gestações foi associado a uma percepção tardia dos pais com relação aos sintomas e ao atraso na primeira avaliação, bem como ao maior intervalo entre elas. Os sintomas motores foram os mais comuns nas crianças mais novas, e as queixas de linguagem nas mais velhas.

## INTRODUCTION

Child development (CD) is a process that prepares children to meet their own needs and the environment’s. It encompasses physical, neurological, social and emotional aspects.^1^ Variations in the development configure distinct and chronic conditions, known as neurodevelopmental disorders.^2^

About 52.9 million of children under the age of five have developmental disabilities, with 95% living in low and middle-income countries.^3^ Among risk factors are biological and environmental components, as well as clinical complications during pregnancy or early life.^4^

For learning purposes, child development can be divided into function domains, which act together: motor (fine and gross), language (verbal and non-verbal), cognition, personal-social and adaptative.^2^ Impairments can occur in one area only, or affect more than one at a time. The latter case is known as Global Developmental Delay (GDD) if the child is under five, or Intellectual Disability (ID) if older, as described in the Diagnostic and Statistical Manual of Mental Disorders (DSM–5).^5^

The early detection of risk factors and developmental problems can provide faster intervention and, thus, enable a better prognosis.^1^ Hence, this study aim was to analyze the profile of patients referred to a specialized outpatient clinic in Brazil. Specifically, the objective was to analyze the epidemiological and clinical profile of the patients assisted in this clinic and to verify factors associated with the child’s age by the time parents first noticed the symptoms and at the first consultation, besides the time interval between the perception of some problem and the consultation in the clinic.

## METHOD

This is a descriptive study, based on retrospective analysis of records of the patients’ first assessments at the Clinic for Integral Attention to Children with Developmental Disorders, in São Paulo, Brazil, between 2009–2018. It was approved by the university’s Research Ethics Committee under number 4.358.99.

This clinic assists children between 0–18 years old, referred from other outpatient units of the same institution, as well as from inpatient care at the university hospital. The service is provided by the Division of General and Community Pediatrics at Escola Paulista de Medicina of the Federal University of São Paulo. The work team is composed of pediatricians, geneticist, physical therapist, psychologist, speech therapist and pediatrics residents. Medical history and parents’ perception of the child’s development are obtained by interview. Child’s development is assessed through observation of language abilities, social interaction and play skills, as well as motor physical examination. The cases are discussed within the group and a diagnostic hypothesis is defined according to the tenth edition of the International Classification of Diseases (ICD-10), and afterwards, a therapeutic plan is drawn.^6^

This sample was obtained by convenience and consisted initially of 383 patients. The inclusion criteria were: children aged 0–18 years, first evaluated between 2009–2018, and referred because of developmental concerns. Patients with missing data, or out of the age and time periods established, were excluded. Thus, nine individuals were removed.

The sociodemographic variables were evaluated: sex, age, address, maternal education and relationship between parents. Regarding pregnancy and childbirth, the following were assessed: whether pregnancy was planned and/or desired, prenatal care, number of previous pregnancies, substance use during pregnancy, gestational age at birth, birth weight, and age at discharge from the nursery. Concerning the child’s clinical condition, their pathological history was assessed, along with the age at which parents first noticed the developmental difficulties, the type of impairment (delay in one of the areas: motor, language, cognition or social, learning disabilities or behavioral disorders) and the diagnostic hypothesis according to the ICD-10.^6^

The main outcomes considering the time of perception and disability assessment were:

1. Types of disabilities and their distribution according to patients’ ages;
2. Children’s ages by the time parents noticed the symptoms;
3. Children’s ages at the first consultation in the clinic; and
4. Time interval between the two dates, as well as the factors associated with them.

Linear regression models were performed to evaluate the associations between variables. The independent variables chosen were maternal education stages, number of previous pregnancies and types of developmental impairment. The dependent variables were child’s ages when the parents noticed the first symptoms and at the first consultation, and the time interval between them. A p-value<0.05 was considered as significant.

## RESULTS

Medical records of 374 individuals were evaluated. Of these, 66.3% were male and 33.7% were female (Table 1). Among them, 72% lived in the state’s capital, mainly in the South (35%) and Southern Central (19%) zones. Only 31% of the districts of origin had specialized rehabilitation centers.

**Table 1.** Demographic characteristics of the 374 children evaluated in the specialized clinic (2009–2018).

Category	n	%
Sex		
Male	248	66.3
Female	126	33.7
Maternal education		
Illiteracy or incomplete elementary school (≤9 years of study)	78	20.9
Complete elementary school (>9 and ≤12 years of study)	78	20.9
Complete high school (>12 and ≤15 years of study)	164	43.9
Complete higher education (15 or more years of study)	33	8.8
No information	21	5.6
Stable relationship between parents		
Yes	243	65.0
No	89	23.8
No information	42	11.2
Desired pregnancy		
Yes	237	63.4
No	45	12.0
No information	92	24.6
Planned pregnancy		
Yes	125	33.4
No	209	56.0
No information	40	10.6
Prenatal follow-up		
Yes	345	92.2
No	15	4.0
No information	14	3.7
Substance use during pregnancy		
No	299	79.9
Alcohol	29	7.8
Smoking	23	6.1
Illicit drugs	16	4.3
Medication	4	1.1
No information	20	5.3
Gestational age at birth		
Preterm	66	17.6
Post-term	4	1.1
Full-term	286	76.5
No information	18	4.8
Birth weight		
<1000g	10	2.7
1000–1500g	10	2.7
1500–2500g	68	18.2
2500–3999g	248	66.3
>4000g	14	3.7
No information	24	6.4

Concerning maternal education, there was a predominance of complete secondary education (43.9%), equivalent to 12 years of study, followed by incomplete elementary education (20.9%), with less than 9 years of study, and finally, 8.8% had 15 years or more. A linear regression model with multiple independent variables did not show association between maternal education and child’s age at parents’ perception of symptoms or at the first assessment. However, the higher the maternal education, the shorter the interval between recognition and clinical assessment (Table 2). Regarding the parents’ civil state, 65.0% were in a stable relationship.

**Table 2.** Linear regression model for the interval between the child’s age when the parents noticed the symptoms and at the first assessment.

Maternal education	Beta	95%CI	p-value
Illiteracy or incomplete elementary school	—	—	—
Complete elementary school	-11.66	-21.88 -1.43	0.025
Complete high school	-12.25	-21.17 -3.34	0.007
Complete higher education	-14.91	-27.99 -1.82	0.025

95%CI: 95% confidence interval.

The median number of previous pregnancies was 1.0, and interquartile range (IQR) 0–2. A linear regression model was performed and showed that the greater the number of previous pregnancies, the older the child’s age when the parents noticed the developmental problems and at the first clinical assessment. Also, the greater number of pregnancies was associated with a greater interval between perception by parents and clinical assessment.

Prenatal care was provided for 92.2% of mothers. Families reported that 56% of pregnancies were unplanned and 63.4% were reported as desired. During gestation, 1.1% of mothers reported the use of some medication (two used isotretinoin), 7.8% reported alcohol intake and 4.3% illicit drugs consumption. The use of substances was either isolated or accompanied by other substances. Children with full-term births prevailed (76.5%). Regarding birth weight, 66.3% had between 2,500–3,999g. The reported age at neonatal discharge ranged from one day to 14 months of life, with a median of three days (IQR 2–9). Of the 374 children, 88% had a positive history of comorbidities or health complications at some point in life, the most frequent being neonatal jaundice (20.4%), and the need for neonatal resuscitation (16.6%) — defined as positive pressure ventilation, chest compressions and/or adrenaline use at birth (Table 3).

**Table 3.** Most frequent previous morbidities in the 374 children evaluated in the specialized clinic (2009–2018).

	n	%
Neonatal jaundice	76	20.4
Neonatal resuscitation*	62	16.6
Pneumonia	47	12.6
Hearing impairment	35	9.4
Asthma	35	9.4
Bronchiolitis	34	9.1
Congenital heart defects	30	8.0
Visual impairment	28	7.5
Epilepsy	26	7.0
Allergic rhinitis	25	6.7
Gastroesophageal reflux disease	24	6.4
Nephropathy	23	6.1
Neonatal sepsis	22	5.9
Amygdala or adenoid problems	21	5.6
Other central nervous system morphological anomalies	20	5.3
Congenital lower urinary tract disorders	19	5.1
Urinary tract infection	19	5.1
Congenital orthopedic problems	16	4.3
Phenotypic deviations	16	4.3

* Neonatal resuscitation is defined as positive pressure ventilation, chest compressions and/or adrenaline use right at birth.

**Figure 1.**
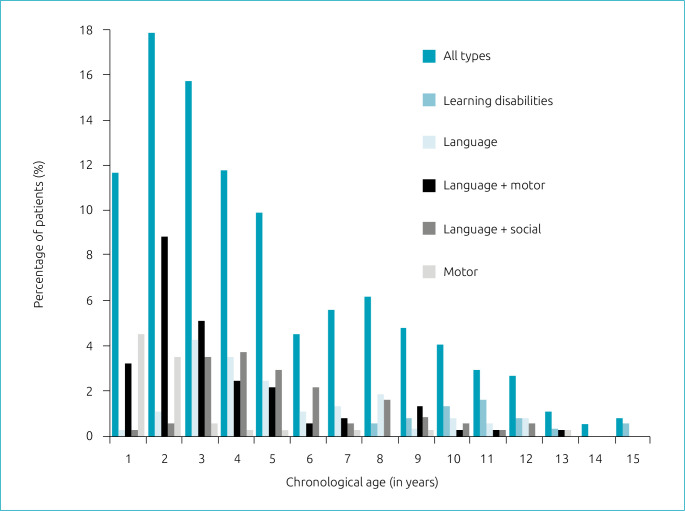
Percentage of the five main types of impairments found, regarding the age at the first assessment.

The patients’ mean age at first consultation was 52.2±39.7 months, with a median of 39.7 months (IQR 19.5–80.6). As described in Figure 1, two peaks of the most prevalent age groups were observed. The first occurred between the 1^st^–4^th^ years, with a predominance of isolated motor delay, as well as language problems associated with motor delay. However, over time, isolated language problems and the combination of disorders in social and language domains became more common and, in the fourth year, they exceeded the other problems. The second peak occurred between the 6^th^–9^th^ years, when language difficulties were more prevalent, whether or not associated with motor or social challenges. A linear regression model with multiple independent variables showed association between the type of disability and the age of the assessment. Children with motor delay would come 22.4 months earlier, while the ones with language problems would come 20.9 months later. Table 4 shows the types of developmental impairments identified.

**Table 4.** Areas of current developmental impairments identified in 374 children evaluated in the specialized clinic (2009–2018).

Areas of developmental impairment	n	%
Language and motor	106	28.3
Language	73	19.5
Language and social	69	18.4
Motor	41	11.0
Language, motor and social	37	9.9
Learning disabilities	25	6.7
Social	2	0.5
Behavior problems	2	0.5
Language and learning disabilities	2	0.5
Language, motor and learning disabilities	1	0.3
Learning disabilities and behavior problems	1	0.3
Normal development	15	4.0
Total	374	100

The mean age at which developmental disorders were first perceived by parents was 20.9±23.8 months, with a median of 12 months (IQR 4.9–23.9). The mean interval until the first consultation was 31.3 months (SD 30.7). An association was also found regarding these variables and the patients’ impairment pattern. Motor delay was predominant in younger children, and language problems were more prevalent in the older ones.

Amid children born preterm, the most prevalent delays were language and motor (30.3%), followed by language and social (22.7%). The parent’s perception of these problems occurred with a mean of 22.7±29.9 months and a median of 9 months (IQR 4.0–23.9).

**Figure 2.**
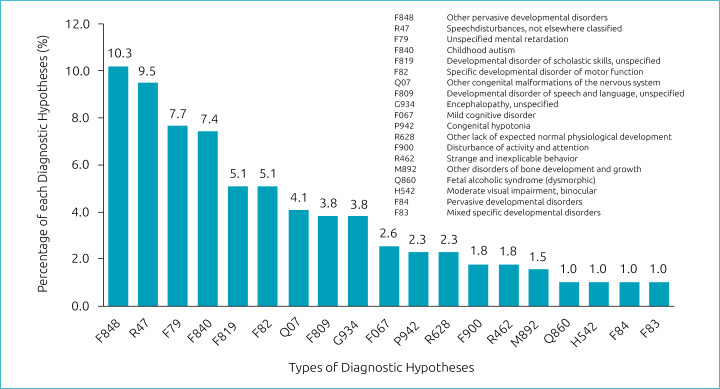
Diagnostic hypotheses with more than 1% of occurrence, with their respective ICD-10 codes.

Among all patients, 359 had a current development disability. Five children had previously solved delay: three with motor and two with language problems. Finally, ten individuals did not show any problem. Considering all 374 patients, 41.9% had GDD, while 15% had ID. Figure 2 shows the main diagnostic hypotheses.

## DISCUSSION

The interval between parents’ perception of symptoms and children’s first consultation was associated with maternal education and number of pregnancies. There was an association between the type of disability and the child’s age at the assessment. Motor symptoms were the most prevalent in earlier ages and language complaints in older children. The number of pregnancies was also associated with the age at which parents noticed the symptoms and at the first consultation. The diagnosis of developmental disorders aims to provide early intervention, in order to improve the patient’s quality of life. However, in most of our patient’s districts, there are no rehabilitation centers available and, therefore, the access to therapies is limited.

In our study there was a male predominance, as in similar studies in Hong Kong and Canada.^7,8^ This may be due to cases of Autistic Spectrum Disorder (ASD), which is more prevalent in boys, as well as X chromosome disorders such as Fragile X Syndrome (FXS), that is responsible for 2–3% of GDD or ID male cases with undefined etiology.^5,9^

As in Singapore and Hong Kong, most mothers had completed high school education.^7,10^ Maternal education is a protective factor for child development, and it is expected that children of mothers with fewer years of schooling are more exposed to risk factors and have less access to interventions.^4^ In this study, the higher the maternal education, the shorter the interval between the parent’s detection of developmental delays and the first specialized assessment.

A stable relationship between parents was mostly reported. Studies show that parents’ emotional stress and impaired mental health may predispose children’s behavioral and developmental problems.^11^

A desired pregnancy (63.4%) was reported by most families assisted in the clinic, although 56% were not planned. This latter condition is associated with a higher prevalence of maternal postpartum depression, which may hinder the mother-baby bonding and, consequently, may be a risk factor for child development.^12^

Most mothers had one previous pregnancy or none. Surprisingly, this study found that the greater the number of previous pregnancies, the older were the child when the parents noticed the disabilities and at the first assessment, and the greater the interval between parents’ perception and evaluation of the child. This could be justified by the small number of patients in our sample. Nonetheless, a Turkish study also found a weak association between lower number of children in the family and higher maternal knowledge of the child’s development. They speculated that mothers with fewer children spend more time with them and may pay more attention to their development.^13^

Prenatal care occurred in most cases. This follow-up helps to identify issues that may potentially be harmful to child development; therefore, low adherence to prenatal care is a risk factor.^14^

Substance use during pregnancy was denied by most parents. In the remaining cases, there was a predominance of alcohol use and smoking, followed by the use of illicit drugs. The first can result in the fetal alcohol spectrum disorders (FASDs).^15^ Likewise, nicotine has a dose-dependent relationship with the occurrence of attention deficit hyperactivity disorder (ADHD).^16^ Furthermore, intrauterine exposure to marijuana is associated with impaired executive functions in adolescence, while cocaine is related to difficulties in sustained attention and behavioral self-regulation.^17,18^ Regarding medication use, it is known that isotretinoin has a teratogenic effect in the central nervous system.^19^

Although prematurity, low birth weight and prolonged length of stay in neonatal intensive care units are risk factors for developmental disorders, most of our patients were born full term (echoing results of a Singaporean center), with a birth weight between 2500–3999g, and they discharged with a median of three days after birth.^10^ This can be explained by the presence, in our institution, of an outpatient clinic dedicated to the follow-up of preterm and very low birth weight children. Therefore, they are not assisted by our team. Still, our data shows a prematurity rate of 17.6%, higher than that of Brazilian population, assessed through the Information System on Live Births (Sinasc) between 2012 (10.87%) and 2019 (9.95%), which points out the importance of the developmental surveillance of these children.^20^

Among the patients’ morbidity history, neonatal jaundice was the most common complication. However, even though this is a reference center, there was only one case of chronic encephalopathy caused by hyperbilirubinemia. Neonatal resuscitation was also frequently reported, and perinatal asphyxia has been associated with neurodevelopmental disorders such as ASD and cerebral palsy.^21^ Other common morbidities were pneumonia, bronchiolitis and asthma. Such conditions are very prevalent during childhood, so it is expected to be reported by families.^22,23^ Congenital heart defects, a group of diseases that may be associated with altered brain structure and executive functions, have also been described.^24^ Auditory and visual problems were also referred. The former may impair language and social development, as well as school performance, and can be a differential diagnosis for ASD or ID.^1,3,25^ Likewise, visual difficulties may affect motor and personal-social development.^25^ Lastly, epilepsy was reported. This can be both a comorbidity of a developmental disorder or its cause. In the first case, there is a higher incidence of epilepsy in patients with ASD. In the second scenario, epilepsy may influence the occurrence of ADHD, in addition to other behavioral challenges.^3^

Glascoe et al. identified high sensitivity and specificity in developmental assessment by parents.^26^ In our practice, the mean age of children by the time their parents realized the symptoms, was 20.9 months, in contrast to the mean age at first consultation that was 52.2 months. This highlights an important delay until the child receives a specialized assessment and, consequently, initiates the intervention, which has better results if started early, due to the intense neuroplasticity in the first years of life.^25^ A similar pattern was observed in Canada, where the average of parental perception occurred on 22.9 months, with arrival at specialized clinics at 38.2 months. There was an interval of approximately 15 months, whereas in our study this interval was of 31.3 months.^8^

Although family concerns started in a timely manner, the long wait for a specific assessment impairs early intervention. Although they are not related to the moment when parents noticed the impairments, social variables can be associated with delay in diagnosis and treatment.^27^ A previous Brazilian study showed that, even though caregivers would seek help early, their developmental concerns were frequently dismissed by pediatricians, which contributed to a greater delay in intervention.^28^

GDD and ID were identified in 56.7% of individuals. Similarly, an Indian center described them in 68.64% of its cases.^29^ The first condition was the most reported, with 41.87%, compared to Canadian and Iranian services, which pronounced it in 35.7% and 54.5% of their patients, respectively.^8,30^ Regarding isolated forms of developmental difficulties, 11% had exclusive motor problems. Other studies have shown different values, such as 29.7% in Iran and 1.2% in India.^29,30^ In our study, 19.5% of children had isolated language disabilities. Likewise, the Singaporean clinic found this in 15.5% of its cases, while a unit in Iran reported 15.6% of its patients.^10,30^

Figure 1 shows that patients with motor difficulties, whether isolated or associated with other challenges, arrived more quickly at the clinic, and it was one of the most prevalent problems in children under two years of age. Language delay, exclusive or related to other difficulties, is presented in all age groups; however, it gains greater prominence from the age of three. This can be explained by motor impairments being more noticeable by pediatricians than language ones.^1^ It is also assumed that, in the latter case, the age at which this is most evident corresponds to the beginning of schooling, when communication skills become more necessary. Exclusive social difficulties were found in 0.5% of cases, similarly to the study in Singapore, in which this was the referral motive in 1% of those evaluated.^10^ Learning disabilities were the only impairment in 6.7% of patients. Other services presented varied data, such as centers in Singapore and India, which showed the condition in 3.8% and 0.4% of cases, respectively.^10,29^ In 0.5% of the patients, isolated behavioral challenges were identified. A survey in the Singaporean clinic described these in 3.1% of cases.^10^ In contrast, they were reported in 18.4% of cases in the Indian center.^29^ According to DSM-5, developmental coordination disorder occurs in 5 to 6% of children aged between 5–11 years.^5^ This diagnosis, corresponding to the “specific developmental disorder of motor function” in ICD-10, was described in 5.1% of our patients.^6^

“Childhood autism”, as established by the ICD-10, was reported in 7.4% of the children. Evaluations from other services show a prevalence of ASD in 18.7%, 22.3% and 23.6% of cases, in India, Canada, and Hong Kong, respectively.^7,8,29^ ADHD was identified in 1.8% of our patients. In comparison, it was reported in 4.5% of the Indian sample and in 5.6% of the assessments of the Singaporean center.^10,29^ However, since in the present study only first evaluations were considered, it is possible that this number may increase as the investigation proceeds.

In this research, it was analyzed the data from a specialized clinic. Nevertheless, it cannot be considered representative of the population of São Paulo or Brazil. Measurement bias must be taken into account, since information was collected by different professionals over the years and subject to their personal interpretation. Another limitation is that standardized tests were not applied and developmental impairments were defined based on parents’ information, as well as child’s physical exam and behavioral observation.

This study described a sample of Brazilian patients with developmental disabilities. In most cases, the parents’ perception of symptoms occurred in an adequate timing, however, there was a delay until the specialized assessment of the child. Higher maternal education was associated with a shorter interval gap. Furthermore, a greater number of previous pregnancies was associated with a later age in the parents’ perception and at the first consultation, as well as a longer interval between them. Motor problems were identified earlier, while language complaints were the most prevalent in older children. These findings suggest that improvement in the care is possible. We hope that these considerations may be useful for similar services and contribute to a better management of children with development disorders in our country.

## Data Availability

The database that originated the article is available with the corresponding author.
